# Systematic reporting to improve the emergency medical response to major incidents: a pilot study

**DOI:** 10.1186/s12873-018-0153-x

**Published:** 2018-01-24

**Authors:** Hardy Sophie, Fattah Sabina, Wisborg Torben, Raatiniemi Lasse, Staff Trine, Rehn Marius

**Affiliations:** 1 Emergency Department, St George’s Hospital, Tooting, London, UK; 2 The Norwegian Air Ambulance Foundation, Drøbak, Norway; 3 Anaesthesia and Critical Care Research Group, Faculty of Health Sciences, University of Tromsø, Tromsø, Norway; 4 Hammerfest Hospital, Department of Anaesthesiology and Intensive Care, Finnmark Health Trust, Hammerfest, Norway; 5 Norwegian National Advisory Unit on Trauma, Division of Emergencies and Critical Care, Oslo University Hospital, Oslo, Norway; 6 Centre for Pre-Hospital Emergency Care/ FinnHEMS 50, Oulu University Hospital, Oulu, Finland; 7 Anaesthesia Research Group, MRC, University of Oulu, Oulu, Finland; 8 Paramedic Sciences, Oslo and Akershus University College, Oslo, Norway; 9 Department of Health Studies, University of Stavanger, Stavanger, Norway; 10 Division of Emergencies and Critical Care. Department of Anaesthesia, Oslo University Hospital, Oslo, Norway

**Keywords:** Major incident, Disaster medicine, Uniform reporting, Standardised data

## Abstract

**Background:**

Major incidents affect us globally, and are occurring with increasing frequency. There is still no evidence-based standard regarding the best medical emergency response to major incidents. Currently, reports on major incidents are non-standardised and variable in quality. This pilot study examines the first systematic reports from a consensus-based, freely accessible database, aiming to identify how descriptive analysis of reports submitted to this database can be used to improve the major incident response.

**Methods:**

Majorincidentreporting.net is a website collecting reports on major incidents using a standardised template**.** Data from these reports were analysed to compare the emergency response to each incident.

**Results:**

Data from eight reports showed that effective triage by experienced individuals and the use of volunteers for transport were notable successes of the major incident response. Inadequate resources, lack of a common triage system, confusion over command and control and failure of communication were reported failures. The following trends were identified: Fires had the slowest times for several aspects of the response and the only three countries to have a single dialling number for all three emergency services had faster response times. Helicopter Emergency Medical services (HEMS) were used for transport and treatment in rural locations and for triage and treatment in urban locations. In two incidents, a major incident was declared before the arrival of the first Emergency Medical Services (EMS) personnel.

**Conclusion:**

This study shows that we can obtain relevant data from major incidents by using systematic reporting. Though the sample size from this pilot study is not large enough to draw any specific conclusions it illustrates the potential for future analyses. Identified lessons could be used to improve the emergency medical response to major incidents.

## Background

Major incidents are a global issue that occur regardless of region or population affected. According to the Centre of Research on the Epidemiology of Disasters (CRED), disasters have become more frequent over the last 20 years [1]. Given the global and humanitarian impact of major incidents, learning lessons from them is essential. However, a Dutch study from 2010 that looked at reports from five consecutive national disasters suggests that we are not learning from them. It observed that, despite changes in protocol, legislation, organisation and funding, the same mistakes were being made each time [2]. Several studies have attempted to determine gold standards for various aspects of the emergency medical response to major incidents, but none have so far been successful [3, 4]. A failure to document, share and learn from our experiences of major incidents may be to blame [5, 6].

A literature review conducted on papers on emergency planning published between 1990 and 2010 found that the majority of publications came from North America and that given the large number of incidents that have occurred in Europe and Australasia, surprisingly few had been published from these areas. It concluded that the validity and generalisability of published literature on emergency planning has not been used to inform policy or change practice and that the type of evidence that would be useful to emergency planners in this respect needs to be identified [7].

Although a structured approach for responders exists [8], recent case reports on major incidents suggest that there is still much room for improvement, particularly in the areas of triage, treatment and transport of patients [9–11], communication [9, 12–14] and sufficient training of emergency medical responders [11].

**Fig. 1:**
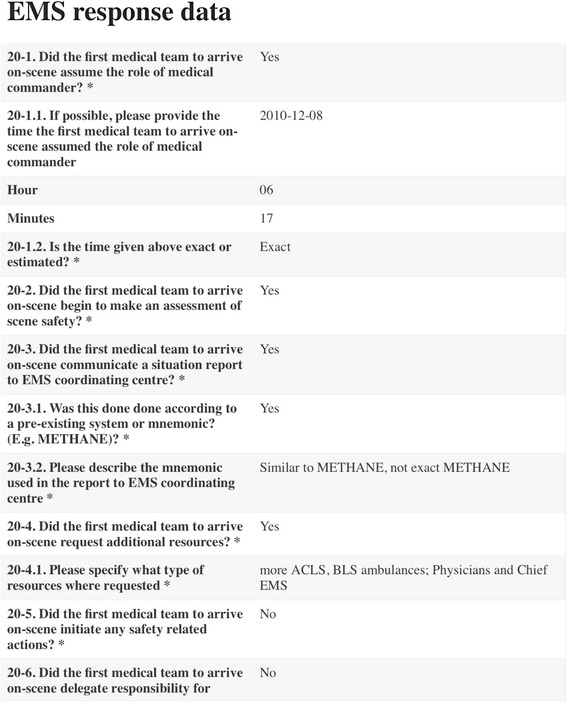
The majorincidentreporting.net consensus-based reporting template

Lately, many papers have highlighted the need to standardise research in this field so that a body of evidence can be assembled from the literature [5, 13, 15–18]. One way to approach this dilemma is to introduce a standardised method of reporting on major incidents (Fig. 1). Templates for reporting on major incidents have been developed globally, but none have been implemented or undergone feasibility testing [19]. This pilot study aims to evaluate whether common perceived successes and failures in major incident medical responses may be identified using a standardised template. A second aim is to investigate if this data can be used to identify trends in the major incident medical response and possible associations between actions and outcome.

## Methods

In the majorincidentreporting.net database, an incident is defined as a major incident if it requires the mobilisation of extraordinary EMS resources and is identified as a major incident in that system [http://majorincidentreporting.net].

**Fig. 2:**
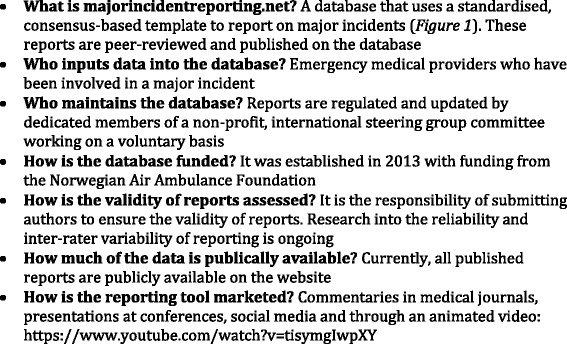
Information on majorincidentreporting.net

This was a retrospective pilot study based on an open access database available on http://www.majorincidentreporting.net [20, 21] (Fig. 2). Data was requested from EMS responders who were involved in a major incident and who had registered their data directly onto our database. Of the eight published major incident reports, the following themes were analysed: time from the occurrence of the major incident to declaration of a major incident, time from the occurrence of a major incident to the arrival of the first EMS, time from the occurrence of the major incident to the initial communication between emergency services, time from the arrival of the first EMS to the time that the major incident was declared, emergency contact number and response times, triage category numbers, effectiveness of communication, command and control and transportation. Whenever possible, these themes were compared between reports.

### Data analysis

Quantitative data from each report was exported onto an Excel spreadsheet version 12.3.6 (©Microsoft Corporation, USA) and presented as frequencies with median and inter-quartile range (IQR). This was used to identify trends that, if consolidated with statistical data from a more significant number of reports, could change the way we respond to major incidents.

**Fig. 3:**
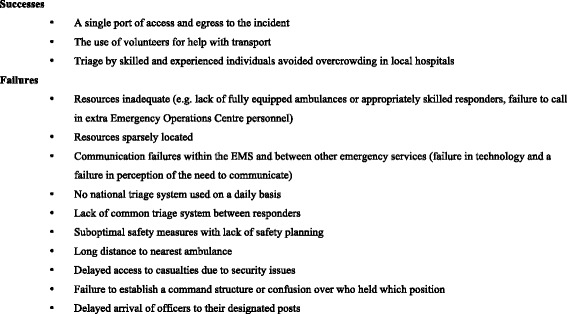
Successes and failures reported in published reports

Perceived successes and failures were extracted as learning points in Fig. 3 to illustrate the potential of the database and data.

### Ethics

Ethics approval for registering data on the website was granted by the Norwegian Data Protection Authority. All reports used in this analysis have been guaranteed by the author to comply with local ethics regulations prior to publication on the website [22].

## Results

Eight reports on real life major incidents were published between April 2015 and March 2017 (Table 1). Key lessons in the published reports are summarised in Fig. 3.

**Table 1:** Details of the eight reports submitted to majorincidentreporting.net to date

Incident	Country	Type of incident	Environment	Number of people at risk
Prison fire	Chile	Fire	Urban	1900
School shooting	Finland	Shooting	Urban	200–299
Bus rollover	Norway	Transport accident	Rural	20
Truck & tunnel fire	Norway	Transport accident Fire	Rural	50
Terrorist mass-shooting	Norway	Shooting	Rural	600–699
Train collision	Norway	Transport accident Fire	Rural	86
Road traffic accident	United Kingdom	Transport accident Extreme weather	Rural	200–299
Hospital gas explosion	Mexico	Gas explosion	Urban	100–199

### Time intervals

A summary of important time intervals is presented in Table 2*.* Mexico, England and Finland were the only three countries in this study to have a single dialling number for all emergency services. Also, the reports from these countries represented the shortest time interval between the major incident being declared and the arrival of first EMS vehicles (6 min, 11 min and 12 min respectively). They also have the shortest time interval from the major incident being declared to the reported time of initial communication between different rescue organisations (1 min, 25 min and 17 min, respectively).

**Table 2:** Time intervals for four aspects of the emergency response with time ranges, median time interval and IQR

Time Interval	Range (min)	Median (min)	IQR (min)
Major incident occurred to arrival of first EMS	6–95	15.5	11.5–63
Major incident occurred to major incident declared	16–72	51	25–66
Major incident occurred to time of initial communication between emergency services	1–90	42.5	17–61
Arrival of first EMS to major incident declared*	10–30	18	15–25

*IQR* Inter Quartile Range

* Does not include results for the two reports in which a major incident was declared before arrival of EMS

A summary of time intervals for each incident report is given in Table 2. In five reports, a major incident was declared after arrival of the first EMS and the time ranged from 10 to 30 min (median 18 min, IQR 15–25 min). In one report, the time interval was missing, and for the remaining two reports, a major incident was declared prior to arrival of the first EMS. One was declared 5 min before and the other 95 min before the arrival of the first EMS.

The two fires, a tunnel fire in Norway and a prison fire in Chile, reported the longest time interval, spanning from occurrence of the major incident to EMS arrival of 49 and 77 min, respectively. Both reports represented the longest time interval from major incident occurred to major incident declared (66 and 72 min., respectively) and the shortest time interval from major incident occurred to time of initial communication between different rescue organisations (61 and 90 min. respectively).

### Triage

**Fig. 4:**
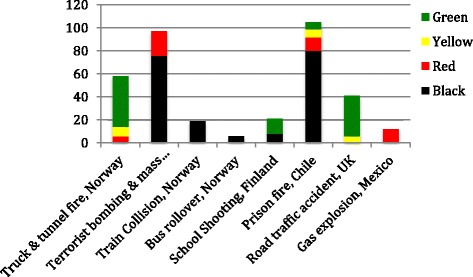
Triage categories for each incident (category numbers marked as “unknown” on the report are not entered)

Triage systems divide casualties into green (delayed), yellow (urgent), red (immediate) and black (dead) categories. Figure 4 depicts triage categories for each incident.

Three reports noted that the triage system used during major incidents was similar to the system they used on a daily basis. In relation to specific failures, three reports cited the lack of a common triage system between responders. In two reports it was highlighted that successful triage by skilled and experienced individuals prevented overcrowding of local hospitals.

### Communication

Failure of communication both within the EMS response teams and between other emergency services (e.g. fire, police, military) was reported in five of the eight reports. Problems included inadequate radio systems with poor coverage, overloading and confusion over choice of communication group and difficulties in communication between two different incident sites resulting in a poor overview of the incident and separate response strategies.

In order to improve the emergency response, introduction of better communication systems with adequate coverage for more personnel and better systems for cooperating with other emergency services, was suggested.

### Command and control

In two reports, confusion over who held which role in the command structure was reported. In one report, delayed arrival of officers to their designated posts was reported to be due to long distances needed to travel. In two reports, they stated a need for more mandatory major incident training or exercises in order to improve MI command and control.

### Transport

In one report, long distances from the incident site to the ambulance loading area was reported as a potential major incident EMS failure. A separate report praised their response for having a single port of access and egress to the incident for ambulances and other emergency vehicles. In two reports the use of volunteers for transporting of patients was suggested as one of their success criteria.

The Helicopter Emergency Medical Services (HEMS) was involved in five out of seven reports. In rural locations, HEMS was used both to transport and treat the patients on scene, while in the remaining reports, HEMS was involved only in triage or treatment on scene.

## Discussion

The results show that we can obtain relevant data regarding common strengths and failures in major incident medical responses by using a consensus based template. Also, these data may be used to identify trends in major incident reporting and identify possible associations between actions and outcome. Moreover, an open-access webpage allows reports to be compared. To our knowledge, no study has been able to analyse a number of standardised reports on different major incidents. Despite the small number of reports, this study shows how results could be analysed to develop and later test hypotheses.

### How results could affect major incident plans

The only three countries to use a single dialling number for EMS, police and fire had the fastest times from occurrence of incident to arrival of first EMS and occurrence of incident to initial communication between different rescue organisations. If later studies show a significant association between these factors, the emergency services should adapt for this.

Compared to the other six reports analysed, the two incidents involving fires took longer to be attended by EMS, longer to be declared as major incidents and longer to establish communication between rescue organisations. If studies later show that there is a positive association between incidents involving fires and longer response times, further studies could be initiated to establish the reason for this and major incident plans could be adapted accordingly.

In most major incident plans, it is the role of the first EMS personnel who arrive on scene to declare or report a major incident [8]. In two out of seven reports, a major incident was declared before the arrival of the EMS. The appropriate declaration of a major incident should occur as early as possible. If later studies prove the hypothesis that declaration of a major incident by the Emergency Operations Centre before the arrival of the EMS is largely appropriate and effective, this information could be used to revise current major incident plans.

### Recurring themes reinforce the need for change in current practice

The findings in this study are supported by others. Failure in communication and confusion over command structure is a recurring theme in major incident case reports [12–14, 23]. Similarly, making use of volunteers especially for transport of patients and resources, the camaraderie or “coming together” of people involved in the incident both from the rescue services and bystanders and their willingness to help beyond their means [24]. This reinforces the concept of “bystander-as-responder”. That bystanders and volunteers should be used more effectively during major incidents and their role should be factored in to major incident plans [25] Also commonly mentioned was the use of highly skilled personnel for triage. This is echoed in a number of publications [23, 26] including a paper by Aylwin et al. which found that overtriage rates were reduced when trained and experienced pre-hospital teams carried out initial scene triage during the London bombings [10].

This study illustrates how a database such as majorincidentreporting.net could facilitate an evidence base for emergency response planning. Studies of a larger number of more homogeneous incidents may provide more valid analysis. Such research initiatives are welcomed.

### Limitations

The number of reports analysed is small and heterogeneous. Additionally, potential subjective bias in the reports cannot be excluded. Most of the published reports have been written by pre-hospital anaesthetists instead of a multi-professional team consisting of paramedics, dispatchers, medical incident commanders and physicians. To overcome this problem, the website could be updated to incorporate a different method of data input so that multiple authors can report on one incident. Furthermore, the reports have been submitted from different countries with different kinds of emergency medical services. Therefore, the comparison of the reports has to be interpreted with caution.

## Conclusion

The findings of this study highlight the importance of identifying strengths and challenges in the major incident medical response through systematic reporting. Further, the identification of trends in the emergency response to major incidents could enable the formulation of hypotheses regarding the best approach to different aspects of the response. Systematic reporting of such data may be used to test hypotheses by comparing data before and after the introduction of new guidelines and policies. Ultimately, it can be used to compare major incidents to determine the optimal medical response. As we move towards a new era of collaborative consumption, anyone can help to develop and learn from a truly global and open access database.

## Abbreviations

ACLS: Advanced Cardiac Life Support
BLS: Basic Life Support
CRED: Centre of Research on the Epidemiology of Disasters
EM-DAT: Emergency Events Database
EMS: Emergency Medical Services
HEMS: Helicopter Emergency Services
METHANE: M-Major incident declared? E-Exact location T-Type of incident H-Hazards A- Access N-Number, Type, Severity of casualties E-Emergency services present and those required
