# Correction to: Systematic reporting to improve the emergency medical response to major incidents: a pilot study

**DOI:** 10.1186/s12873-018-0157-6

**Published:** 2018-02-09

**Authors:** Sophie Hardy, Sabina Fattah, Torben Wisborg, Lasse Raatiniemi, Trine Staff, Marius Rehn

**Affiliations:** 1 Emergency Department, St George’s Hospital, Tooting, London UK; 2 The Norwegian Air Ambulance Foundation, Drøbak, Norway; 3 Anaesthesia and Critical Care Research Group, Faculty of Health Sciences, University of Tromsø, Tromsø, Norway; 4 Hammerfest Hospital, Department of, Anaesthesiology and Intensive Care, Finnmark Health Trust, Hammerfest, Norway; 5 Norwegian National Advisory Unit on Trauma, Division of Emergencies and Critical Care, Oslo University Hospital, Oslo, Norway; 6 Centre for Pre-Hospital Emergency Care/ FinnHEMS 50, Oulu University Hospital, Oulu, Finland; 7 Anaesthesia Research Group, MRC, University of Oulu, Oulu, Finland; 8 Paramedic Sciences, Oslo and Akershus University College, Oslo, Norway; 9 Department of Health Studies, University of Stavanger, Stavanger, Norway; 10 Division of Emergencies and Critical Care. Department of Anaesthesia, Oslo University Hospital, Oslo, Norway

## Erratum

The original article [1] contains an error whereby all authors’ names were mistakenly interchanged. The original article has now been corrected to present the authors’ names correctly.

In addition, this error was mistakenly carried forward by the production team that handled the article, and as such, was not the fault of the authors.

## References

[1] Hardy S (2018). Systematic reporting to improve the emergency medical response to major incidents: a pilot study. BMC Emerg Med.

